# Frequentmers - a novel way to look at metagenomic next generation sequencing data and an application in detecting liver cirrhosis

**DOI:** 10.1186/s12864-023-09861-w

**Published:** 2023-12-12

**Authors:** Ioannis Mouratidis, Nikol Chantzi, Umair Khan, Maxwell A. Konnaris, Candace S. Y. Chan, Manvita Mareboina, Camille Moeckel, Ilias Georgakopoulos-Soares

**Affiliations:** 1 Department of Biochemistry and Molecular Biology, Institute for Personalized Medicine, Penn State College of Medicine, Hershey, PA USA; 2 Bakar Computational Health Sciences Institute, University of California San Francisco, San Francisco, CA USA; 3 Department of Bioengineering and Therapeutic Sciences, University of California San Francisco, San Francisco, CA USA; 4 Department of Statistics, Penn State, University Park, PA USA; 5 Huck Institutes of the Life Sciences, Penn State, University Park, PA USA

**Keywords:** mNGS, k-mers, liver lirrhosis, detection

## Abstract

Early detection of human disease is associated with improved clinical outcomes. However, many diseases are often detected at an advanced, symptomatic stage where patients are past efficacious treatment periods and can result in less favorable outcomes. Therefore, methods that can accurately detect human disease at a presymptomatic stage are urgently needed. Here, we introduce “frequentmers”; short sequences that are specific and recurrently observed in either patient or healthy control samples, but not in both. We showcase the utility of frequentmers for the detection of liver cirrhosis using metagenomic Next Generation Sequencing data from stool samples of patients and controls. We develop classification models for the detection of liver cirrhosis and achieve an AUC score of 0.91 using ten-fold cross-validation. A small subset of 200 frequentmers can achieve comparable results in detecting liver cirrhosis. Finally, we identify the microbial organisms in liver cirrhosis samples, which are associated with the most predictive frequentmer biomarkers.

**Supplementary Information:**

The online version contains supplementary material available at 10.1186/s12864-023-09861-w.

## Introduction

Early detection and diagnosis of human disease is essential for enhancing patient outcomes and reducing mortality rates [1], as it enables timely efficacious intervention strategies. However, current modalities of detecting disease often lack the sensitivity and specificity required to capture presymptomatic stages of disease progression accurately. Therefore, there is an urgent need to establish novel methods that identify unique biological markers of disease which precede the manifestation of symptoms.

Sequencing has provided the opportunity to investigate the molecular insights of disease mechanisms and potentially identify unique changes between healthy and affected individuals. Kmers, contiguous DNA subsequences of length *k*, have been successfully implemented across multiple research problems including in the construction of genome alignments [2], for the generation of genome assemblies [3], in understanding evolutionary relationships between species [4] and the construction of phylogenies [5] among other applications. Additionally, a number of algorithms have been developed for the faster and more efficient derivation of kmers and their occurrences, such as Jellyfish [6] and BBDuk [7]. Kmers have been previously used to describe new features or characteristics of an organism related to the presence or absence of a specific contiguous subsequence. For example, the subset of kmers that do not appear in a genome are referred to as nullomers [8–10] and the subset of kmers that are found in a single species are referred to as quasi-primes [11]. Using kmer strategies, we may efficiently mine the human microbiome for differences that distinguish patients with disease from healthy individuals in effort to establish unique biological signatures.

Liver cirrhosis is a major health burden across countries, affecting 5.2 million people globally and causing 1.48 million deaths in 2019 alone [12]. The proportion of liver cirrhosis deaths as a fraction of total deaths in the population has increased in the last decade, indicating the need for early detection and intervention, including lifestyle changes and treatments [13]. Metagenomic Next Generation Sequencing (mNGS) is a powerful tool that enables researchers and clinicians to identify and characterize microbial pathogens, antimicrobial resistance, and virulence markers from various samples, which can facilitate early disease detection and diagnosis. In a study conducted by Qin et al. [14], a cohort of 123 patients with liver cirrhosis and 114 healthy individuals was studied using mNGS data from stool samples [14]. Qin et al. trained a disease classifier using leave-one-out cross-validation on 98 patient and 83 healthy control samples to identify gene markers enriched either in patients or controls. The computational complexity of this cross-validation approach informed the decision of the authors to only use fifteen gene markers as features for the Support Vector Machine (SVM) model, achieving an AUC value of 0.836. Improvements in the performance of such a model would be required for the clinical implementation of mNGS for liver cirrhosis detection.

Machine learning methods have been extensively used to study mNGS data for signs of a variety of different diseases [15]. Compressed representations of the metagenome (via the FracMinHash algorithm [16] have been used in combination with random forests to study Inflammatory Bowel Disease (IBD) [17]. Long k-mers, ranging from thirty to thousands of base pairs long, have been also used for metagenomic analysis [18, 19]. Here we describe a feature selection approach for machine learning models based on short kmers, trying to capture the most succinct units of information unique to the patient and control cohorts. We refer to these short kmers as that are present in multiple samples from one group but completely absent from the other group as frequentmers, referring to the frequency they appear in their respective cohorts. We extracted frequentmers across the mNGS dataset from the liver cirrhosis study, utilizing them to train a machine learning model that achieves an AUC of 0.91 with tenfold cross-validation. We demonstrated that a small number of 200 frequentmers can result in comparable classification accuracy. We also identified specific microbial species that are informative for the detection of liver cirrhosis. Frequentmers are transferable to other diseases and sequencing assays, representing a novel method for biomarker development and for the detection of human diseases.

## Results

### Derivation of frequentmers

**Fig. 1:**
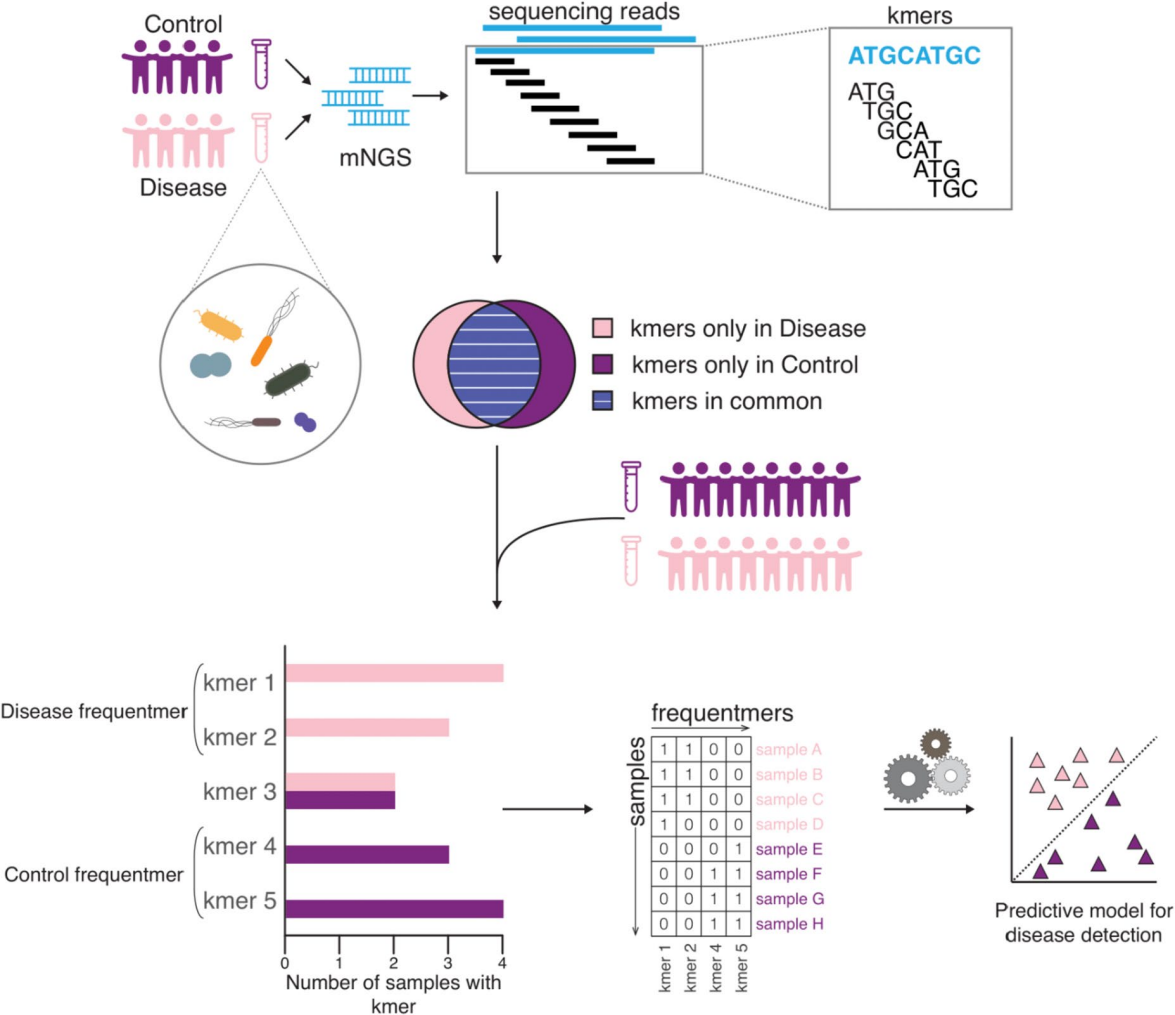
Visualization of frequenter extraction pipeline and inference. Two groups of samples are examined, the first group is composed of healthy control samples and serves as the control and the second group contains patient samples, for the disease that is investigated. mNGS data are analyzed to determine the number of kmers found in each sample and subsequently the kmers unique to only one group (healthy controls or patients) are identified. Frequentmers represent the recurrent kmers found only in patient samples or only in healthy control samples, but never in both. Frequentmers that are found in multiple samples of only one group are used as features to train a machine learning algorithm to perform binary classification on unseen data

We have derived a new type of algorithm that identifies highly informative and specific sequences to enable the early detection of human diseases (Fig. 1). First, we identified the set of kmer sequences that are observed in each patient and each control sample. Next, we calculated the number of samples in which each kmer sequence is present. We removed sequences that are present in both patient and control samples, hypothesizing that these sequences are less likely to reflect differences between the two groups, and to serve as biomarkers. The subset of sequences that are found in multiple healthy control samples and not in patient samples is termed “control frequentmers” and similarly the subset present in multiple patient samples and absent from healthy control samples is termed “patient frequentmers”. The aforementioned process is performed using ten-fold cross-validation. For each fold, 90% of the samples are used as a training set and the remaining 10% is used as a test set. Frequentmers are derived independently from the training set of each fold. The mathematical formulation is provided below.

### Definitions

Let us define alphabet $L = \{A, T, C, G\}$ representing adenine, thymine, cytosine, and guanine respectively. Metagenomic Next Generation Sequencing reads can be represented as a nucleotide string $R={t}_{1}{t}_{2}{t}_{3}...{t}_{z}$ over this alphabet. We can then represent the entirety of an individual’s sequenced metagenome as a collection of strings $I = \{{R}_{1}, {R}_{2}, {..., R}_{n}\} .$

A nucleotide kmer $K$ is defined as a short nucleotide sequence of length k over alphabet $L$ and can be represented as $K = {s}_{1}{s}_{2}{s}_{3}...{s}_{k}.$ A kmer $K$ is said to belong to a read$R$,$k\in {R}_{i}$, if and only if$\exists i,j\in \{1, ..., z\}: j-i =k-1 \wedge {s}_{1}{s}_{2}{s}_{3}...{s}_{k} ={t}_{i}{t}_{2}{t}_{3}...{t}_{j}$. Note that $j-i =k-1$ implies the kmer is comprised of exactly $k$ nucleotides.

A kmer $K$ is said to belong to an individuals metagenome $I$ if and only if $\exists i: k\in {R}_{i} \wedge {R}_{i}\in I$.

The samples used for training our algorithm can further be subdivided into two distinct groups, mNGS sequencing of samples taken from healthy control samples and mNGS sequencing taken from individuals with liver cirrhosis. Let us name the two groups$H = \{{H}_{1}, {H}_{2}, {H}_{3}, ..., {H}_{m}$} and $P = \{{P}_{1}, {P}_{2}, {P}_{3}, ..., {P}_{l}\}$ of controls and patients respectively.

A kmer $K$ is said to be a healthy control frequentmer of recurrency $r$ if and only if:

$$(\exists {a}_{1}, {a}_{2}, {a}_{3}, ..., {a}_{r}\in \{1, ..., m\}: \forall i,j\in \{1, ..., r\} {a}_{i}\ne {a}_{j} \wedge K \in {H}_{{a}_{i}})\wedge (\forall o \in \{1, ..., l\}: K \notin {P}_{o})$$

In other words, a kmer $K$ is said to be a healthy control frequentmer of recurrency *r* if and only if this k-mer appears in at least r control samples and does not appear in any patient samples.

Similarly, a kmer $K$ is said to be a patient frequentmer of recurrency *r* if and only if:

$$\exists {a}_{1}, {a}_{2}, {a}_{3}, ..., {a}_{r}\in \{1, ..., l\}: \forall i,j\in \{1, ..., r\} {a}_{i}\ne {a}_{j} \wedge K \in {P}_{{a}_{i}})\wedge (\forall o \in \{1, ..., m\}: K \notin {H}_{o})$$

In other words, a kmer $K$ is said to be a patient frequentmer of recurrency *r* if and only if this kmer appears in at least *r* patient samples and does not appear in any control samples.

### A disproportionate number of liver cirrhosis-specific kmers is detected

We implemented our algorithm in metagenomic Next Generation Sequencing (mNGS) data derived from fecal samples of liver cirrhosis patients and healthy controls [14]. In total, we examined 123 patients with liver cirrhosis and 114 matched healthy controls. We extracted every sixteen base-pair (bp) kmer found in each sample and split samples in ten groups or folds, with the proportion of cases over the total samples in each fold being maintained. The choice of kmer length was informed from previous studies in which we found that the performance of the kmer-based models increased as a function of kmer length up to sixteen bp length [20]. For each fold, we examined which subset of the total kmers detected constituted frequentmers, using the number of samples in which each kmer was found as the recurrency threshold (see Methods). Thus, we estimated the number of healthy control and patient frequentmers as a function of the recurrence among liver cirrhosis patients and healthy controls, respectively.

**Fig. 2:**
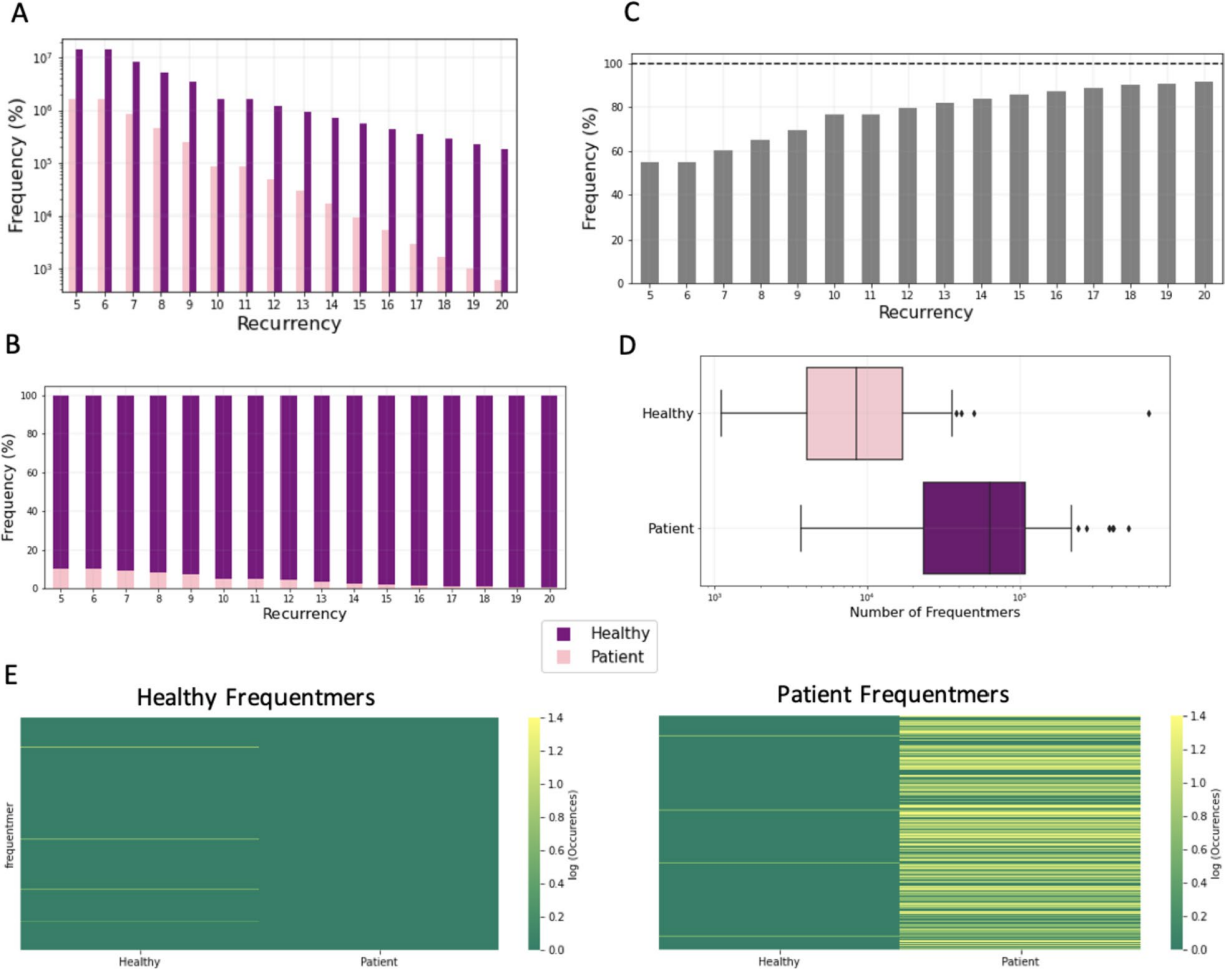
Characterization of frequentmers associated with liver cirrhosis. **A** The number of liver cirrhosis frequentmers and healthy control frequentmers identified as a function of the number of samples in which they were detected (recurrency). **B** Stacked barplot showing the proportion of the total frequentmers being patient and healthy control frequentmers. **C** Bar plot displaying the frequency with which frequentmers identified in the training set are observed in the test set, for recurrency thresholds 5–20. Results shown represent the mean across the ten folds. **D** Number of frequentmers detected in the test set for healthy control and liver cirrhosis frequentmers for recurrency threshold of fifteen across folds. Results shown represent the mean across the ten folds. **E** Number of healthy frequentmers in the training set also detected in the test set of healthy control and patient samples (left). Number of liver cirrhosis frequentmers in the training set also detected in the test set of healthy control and patient samples (right). Frequentmers of the recurrency threshold of fifteen samples were used

First, we find that the number of frequentmers recovered decreases as a function of the recurrency threshold used (Supplementary Fig. 1a-c). We also find that as the recurrency threshold for the number of samples a frequentmer is present in increases, there is a larger proportion of the total frequentmers being patient frequentmers relative to control frequentmers (Fig. 2a-b; Supplementary Fig. 2; Pearson correlation: *r* = 0.975, *p*-value < e-9). Specifically, we observe that for the recurrency threshold of five samples, there is 8.92-fold more patient than healthy control frequentmers, whereas at recurrence of twenty samples there is 306.5-fold more patient than healthy control frequentmers (Fig. 2b; binomial test, *p*-value = 0), indicating an imbalance between the number of healthy control and patient frequentmers identified. These differences likely stem from changes in the microbiome of liver cirrhosis patients, which are observed recurrently across multiple liver cirrhosis patients and which are not normally observed in healthy microbiomes.

Next, we examined what proportion of frequentmers observed in the training cohort was also identifiable in the test cohort. We find that the proportion of frequentmers observed in the test cohort is correlated with the recurrence threshold (Fig. 2c; Supplementary Fig. 3; Pearson correlation: *r* = 0.964, *p*-value < 2.03e-9). We also observe that for recurrence threshold of fifteen samples, 86% of frequentmers are recovered, with the proportion of frequentmers that is recovered leveling off around this recurrence threshold (Fig. 2c). Importantly, the number of patient frequentmers detected in the test set is significantly larger in samples from liver cirrhosis patients relative to healthy controls across the recurrency thresholds examined (Fig. 2d; Mann–Whitney U, *p*-value = 0). Additionally, we find that healthy control frequentmers from the training set are 6.49-fold more likely to be found in healthy control samples in the test set (Fig. 2e; Mann–Whitney U, *p*-value < 0.00016). Similarly, liver cirrhosis frequentmers derived in the training set are 9.04-fold more likely to be found in liver cirrhosis samples in the test set (Fig. 2e; Mann–Whitney U, *p*-value < 9.1e-5), providing further support for efficacy of our methodology. Therefore, we find that across the ten folds that were independently evaluated, there are frequentmers that are consistently detected and that are either liver-cirrhosis specific or only derived from healthy control samples.

### Identification of kmers associated with HBV infection and high alcoholic consumption

Liver cirrhosis is linked to both high alcohol intake and HBV infection [21]. For liver cirrhosis, we investigated if there were differences in the samples that are Hepatitis B virus (HBV) positive, regarding the frequentmers detected.

**Fig. 3:**
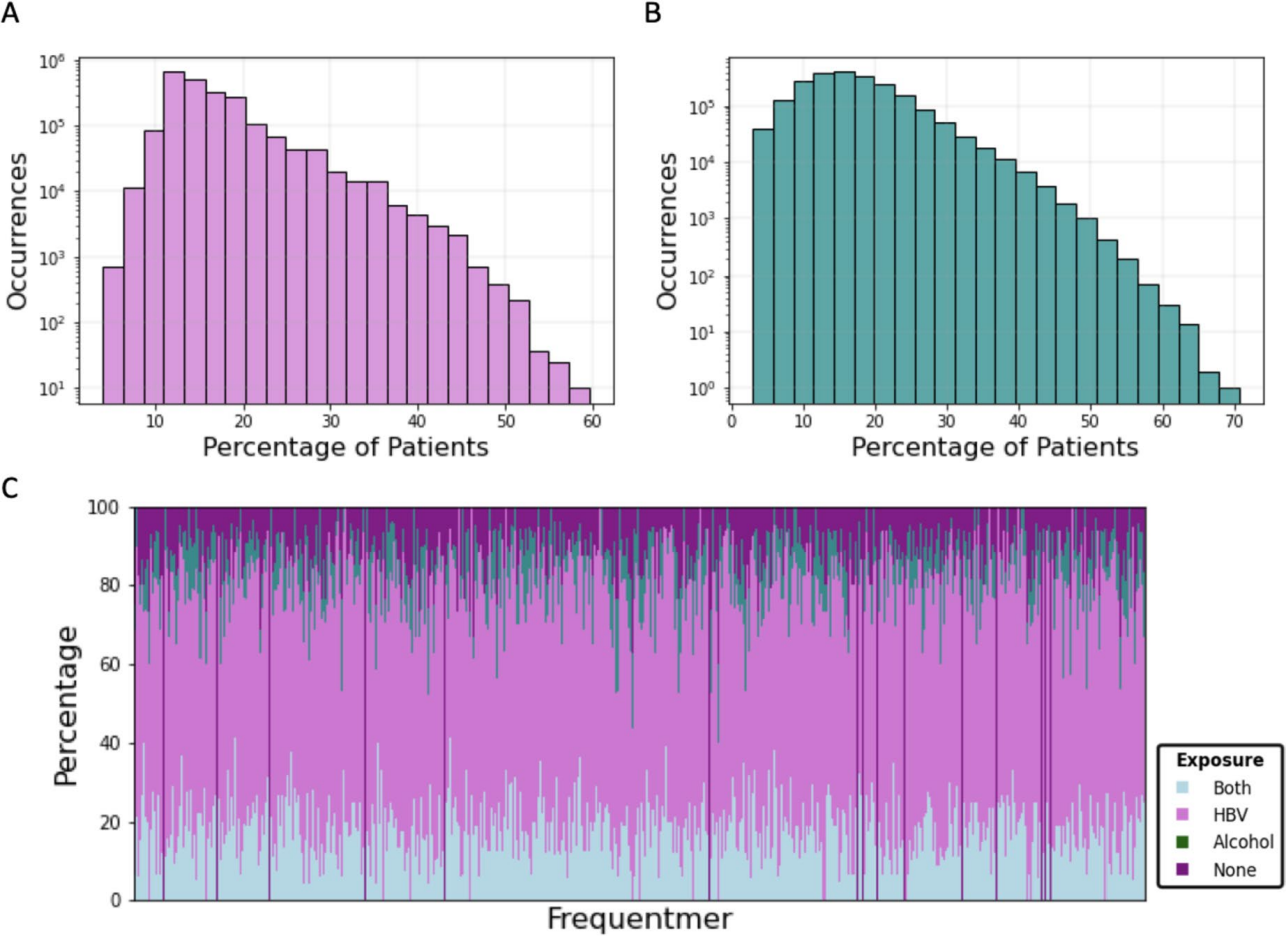
Characterization of liver cirrhosis frequentmers in relationship to HBV infection and alcohol consumption. **A** Histogram displaying the number of frequentmers and the corresponding percentage of HBV-positive patient samples they were detected. **B** Histogram displaying the number of frequentmers and the corresponding percentage of alcohol samples in which they were detected. **C** Frequentmer distribution in samples that are HBV-positive (*n* = 99), have high alcohol intake (*n* = 34), are both HBV-positive and have high alcohol intake (*n* = 23) or are not associated with either (*n* = 13)

Among the liver cirrhosis frequentmers of recurrency fifteen, we find that 55,789 are specific to liver cirrhosis patients that are HBV positive and are completely absent from HBV negative patients, for recurrency (Fig. 3a; Supplementary Fig. 3). Similarly, we examined samples from alcohol-related liver cirrhosis patients and observed that 5,004 are specific to them for recurrency threshold of five (Fig. 3b; Supplementary Fig. 4). The recurrency threshold of five and fifteen were selected as they represent an approximately equal proportion of the total number of samples that are alcohol-related and HBV positive, which are 34 and 99 respectively. Therefore, we conclude that we can detect healthy control frequentmers, general liver cirrhosis frequentmers and frequentmers that reflect viral exposure (HBV) and lifestyle (alcohol consumption) differences. We find that the majority of frequentmers originate primarily from HBV positive samples, which is to be expected as they constitute the majority of our patient samples. However, there are differences in the distribution of frequentmers, with some tending to be found more in either HBV positive or alcohol-related samples (Fig. 3c).

We examined the number of frequentmers of recurrency fifteen shared in HBV positive patients relative to patients that were HBV negative and observed that the two groups were dissimilar in their frequentmer profile (Mann–Whitney U, *p*-value < 0.0143). Similar results were observed for samples that were derived from high alcohol intake patients relative to other patients (Mann–Whitney U, *p*-value < 3.49e-6). We conclude that differences in the exposures of samples are reflected in their frequentmer profile.

### Principal component analysis reflects differences in frequenter profiles

**Fig. 4:**
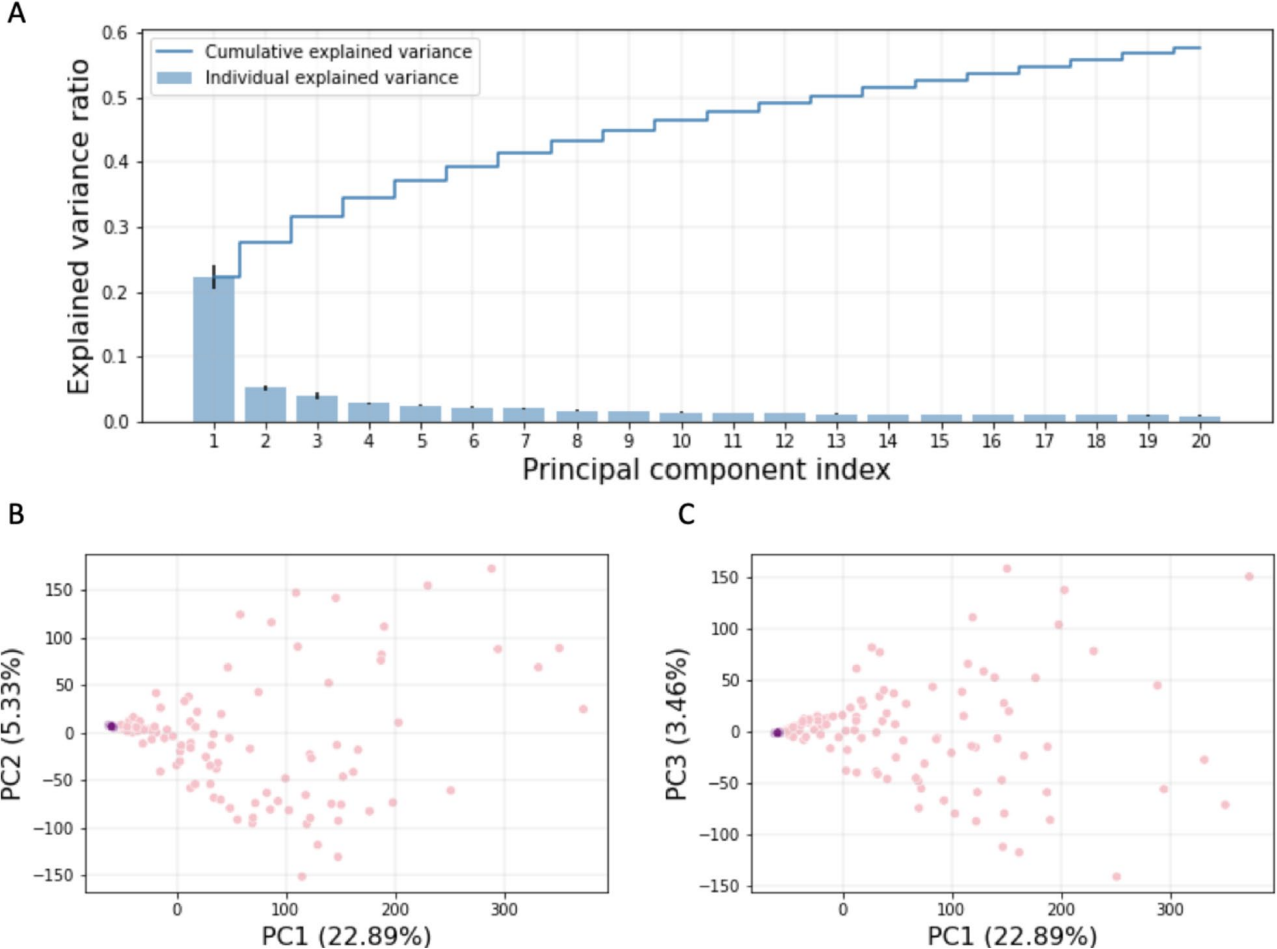
**A** Proportion of variance explained by the first twenty principal components. Mean score of the explained variance ratio across the ten folds is shown. Error bars show standard deviation. Line plot indicates the cumulative explained variance across the twenty first principal components. **B**-**C** Scatter plot displaying the separation of patient and control samples by the first three principal components. Results shown for **B**. PC1 versus PC2 and **C**. PC1 versus PC3

Next, we examined if liver cirrhosis and healthy control samples are linearly separable. A principal component analysis (PCA) was used to examine the information that healthy control and liver cirrhosis frequentmers can capture to separate samples from the two groups. We observe that a large fraction of the variance can be explained by the first twenty principal components (PCs), with the first PC alone capturing 22.89% of the variance (Fig. 4a). Additionally, we observe that the first three PCs can separate the liver cirrhosis and control samples (Fig. 4b-c). These findings provide evidence that frequentmers can capture differences in the mNGS profile of liver cirrhosis patients and healthy controls.

### A predictive model based on frequentmers can accurately detect liver cirrhosis

**Fig. 5:**
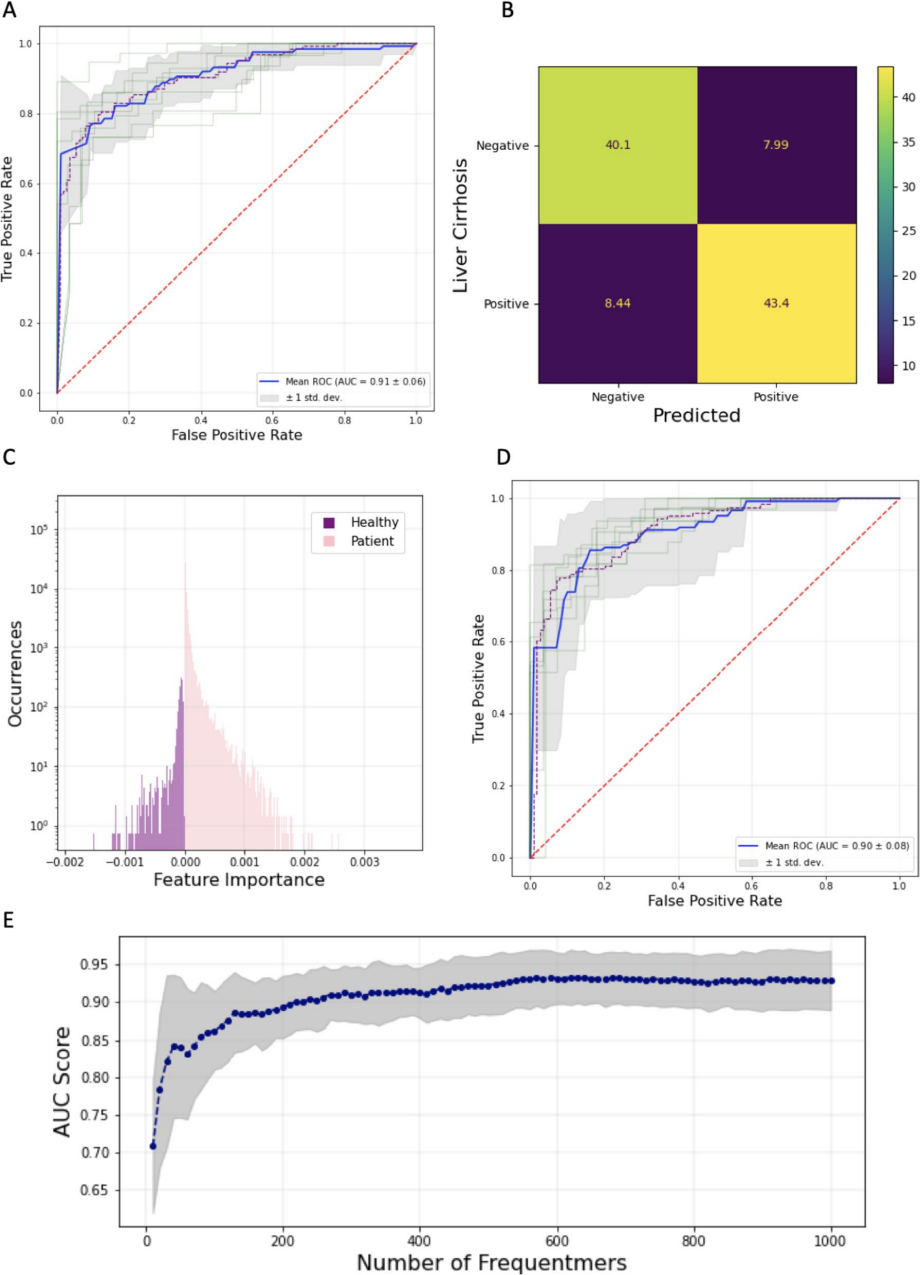
Machine learning based liver cirrhosis detection. **A** ROC curve displaying the AUC for the logistic regression model for recurrency threshold of fifteen. **B** Confusion matrix showing the percentage of samples that were correctly and incorrectly classified as liver cirrhosis patients or healthy controls, for recurrency threshold of fifteen. **C** Logistic regression classification coefficients. **D** ROC curve displaying the AUC for the XGBoost classification model, for recurrency threshold of fifteen. **E** AUC score relative to number of top frequentmers used for logistic regression. Gray lines display the confidence intervals from the ten folds. The blue line shows the mean AUC score across the ten folds

The early detection of liver cirrhosis is critical for intervention and improved clinical outcomes of patients [22]. We therefore developed machine learning classification models to examine if frequentments can accurately predict liver cirrhosis patients from healthy controls. The first model we examined was a logistic regression model, which has inherent advantages such as interpretability and determinism. We examined the performance of the model using multiple recurrency thresholds for the number of samples in which each frequentmer was found in the training set. We observed that when increasing the sample recurrency threshold, the performance of the model increased (Supplementary Fig. 6), which is likely due to removing features that were less informative. We also report that the logistic model has an AUC of 0.91 for recurrency threshold of fifteen samples (Fig. 5a-b), indicating that it can accurately detect liver cirrhosis. The performance of our model was superior to that obtained from the original article [14]. We also find that the top features are liver cirrhosis frequentmers (Supplementary Figs. 7 and 8). From the 1,000 most informative coefficients (as measured by absolute coefficient score) of the logistic regression model 993 were liver cirrhosis frequentmers, which was significantly more than expected by chance (Binomial test, *p*-value < 1.4e-07; Fig. 5c, Supplementary Fig. 7). As a result, we conclude that our feature selection is largely reflected in what the logistic regression model learns and is primarily based on liver cirrhosis frequentmers.

To examine non-linear patterns between frequentmers we also implemented an XGBoost classification model. Extreme Gradient Boosting is a classification framework based on training a sequence of decision trees and utilizing their combined predictions to make the final classification [23]. We observe that across the different sample recurrency thresholds the model performs comparably to the logistic regression model (Supplementary Fig. 9, Fig. 5d). At low recurrency thresholds, for which the number of features is extremely large and the noise in the system increases, XGBoost outperforms logistic regression (Supplementary Fig. 6, Supplementary Fig. 9). However, for a frequentmer recurrency threshold of fifteen samples we obtained an AUC score of 0.90, suggesting that the ensemble model did not perform better than the logistic regression model. We conclude that the model that is based on linear relationships is sufficient to capture the majority of the variance and therefore, due its clear advantage in explainability over the XGBoost model, is our preferred method.

Additionally, we were interested to find if we can perform similarly in detecting liver cirrhosis using only a small fraction of the frequentmers. We therefore investigated how the number of frequentments used in the classification model influenced the performance. To achieve this we identified the most informative features from the training set of each fold in the logistic regression using the absolute value of each logistic regression coefficient for each frequentmer (Fig. 5c) and re-trained the logistic regression model for the same samples in the training set. We then tested the performance of our model. This process was repeated, examining between 25 and 1,000 frequentmers. We observe that even with roughly 200 frequentmers we achieve comparable performance to the original model that used all frequentmers (Fig. 5a, e). These results indicate that with a small number of frequentmers we can be used to generate a classification model that can accurately detect liver cirrhosis.

### Identification of microbial species driving the classification models

Utilizing the coefficients of the logistic regression performed at recurrency threshold fifteen, which corresponded to the best performing model, we identified the 100 frequentmers with the highest positive regression coefficient and the 100 frequentmers with the lowest negative regression coefficient averaged over all ten folds. Within this group, the 100 frequentmers with a positive coefficient were patient frequentmers and the 100 frequentmers with a negative coefficient were healthy control frequentmers.

**Fig. 6:**
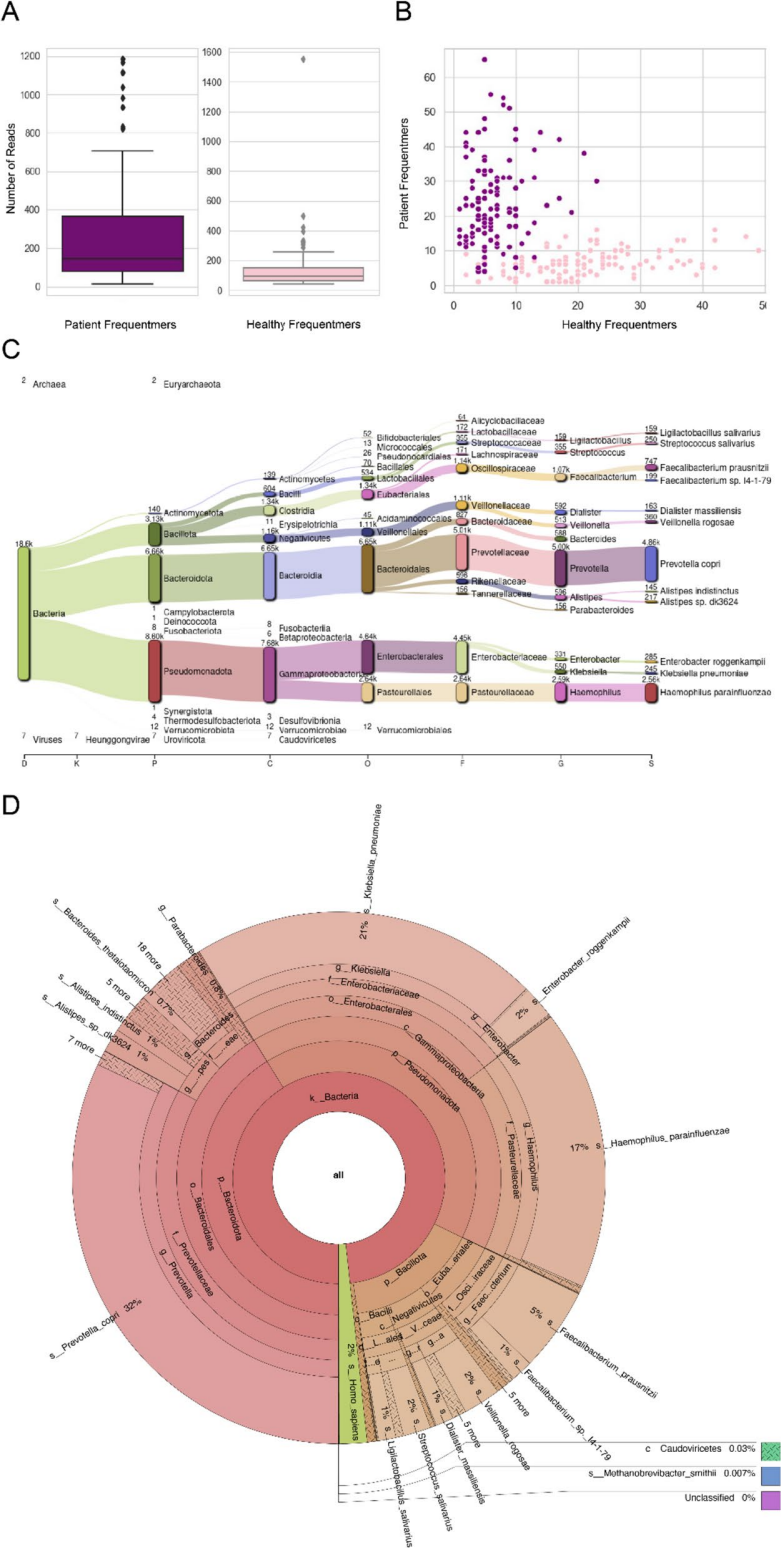
Frequentmer containing reads. Top and bottom 100 most informative frequentmers based on logistic regression with recurrency threshold fifteen. **A** The corresponding number of reads originated from. **B** Their distribution across patient and healthy control samples. **C** Taxonomic assignment for the top frequentmer reads across microbial organisms. **D** Krona RSF plot showing the identified microbial species abundance

We then extracted the sequencing reads from which those frequentmers originated, identifying a total of 41,944 reads. On average, these frequentmers were present in 210 reads (mean: 209.72, standard deviation: 256.50), with some significant outliers skewing the variance (Fig. 6a). Patient frequentmers were found in significantly more reads than healthy frequentmers (t-test, *p*-value < 0.00006). The distribution of samples and the number of patient and healthy frequentmers supports a clear separation between patient and control samples (Fig. 6b).

We further identified the microbial species from which the frequentmers were derived from. Interestingly, we find a set of bacterial species that are highly enriched for frequentmers (Fig. 6c-d), including *Prevotella copri*, *Haemophilus parainfluenzae*, *Faecalibacterium prausnitzii* and *Klebsiella pneumoniae*, multiple of which have been previously associated with liver cirrhosis [24] or other liver-associated diseases such as liver abscess [25], and Nonalcoholic Fatty Liver Disease [26]. Therefore, we conclude that we can identify the microbial species from which the frequentmers were derived, showcasing the interpretability of our approach.

## Discussion

In this work, we describe the development of a method that enables the identification of kmer sequences that are specific to patient and healthy control samples, which we term patient and healthy control frequentmers, respectively. We show that frequentmers can be used as disease detection biomarkers, by demonstrating their utility for the detection of liver cirrhosis using mNGS data, in which we outperform previously published models and achieve an AUC score of 0.91 [14].

The integration of other biomarkers, clinical information and risk factors can result in further improvements of our models for the early detection of human disease. Furthermore, a major strength of our method is the interpretability of our logistic regression model and we provide evidence that we can directly infer the microbial species from which the frequentmers are derived from. We show that the majority of high importance frequentmers originate from microorganisms known to be associated with liver cirrhosis, such as *Prevotella copri*, *Faecalibacterium prausnitzii* and *Klebsiella pneumoniae*, multiple of which have been previously associated with liver cirrhosis [24, 27, 28]. These results demonstrate the ability of the method to discover new associations between microbial species in the gut microbiome and disease. Investigation of the biological function of these microbial species and their roles in liver damage and cirrhosis are of particular interest for future work. Therefore, frequentmers can provide insights into microbial changes specific to the development of liver cirrhosis which could result in mechanistic insights for the role of the microbiome in this disease.

Examination of fecal samples using mNGS data is a non-invasive procedure that can be used for the early detection of liver fibrosis, before the manifestation of symptoms associated with liver cirrhosis. A small set of frequentmers suffices to achieve high predictive power in detecting liver cirrhosis (Fig. 5e), which is another important advantage of our method. As a result, the detection of a small set of frequentmers could enable novel diagnostics based on short DNA sequences from mNGS data. For instance, CRISPR-based detection tools could be used to target frequentmers in detection assays [29] or sequencing-based approaches such as adaptive sampling (also known as selective sequencing), which can enrich for specific sequences [30], can be applied to reduce detection costs.

A number of human diseases are associated with changes in the human microbiome, including cancer [31] neurodegenerative diseases [32], metabolic diseases [33], autoimmune disorders [34, 35] and various infections [36–38]. Therefore, machine learning models based on frequentmers could be applicable towards the detection of multiple other diseases as well as for pathogen detection. Furthermore, our methodology can be transferred across different experimental assays beyond mNGS, and it will be of interest to investigate its utility for cfDNA and cfRNA based diagnostics. The origin of the biological material can also vary and in future work it will be of interest to develop frequentmer based classification models for urine, saliva and blood samples.

In summary, we provide a novel methodology for the derivation of disease detection biomarkers and showcase their utility in the detection of liver cirrhosis from mNGS data obtained from fecal samples. A limitation of this study is its application to the single liver cirrhosis mNGS group. Future work could provide validation of this method in different experimental studies and across additional diseases. Analyzing additional datasets and expanding our findings in a multi-disease detection assay that is based on disease-specific frequentmers, could enable clinical screening applications.

## Methods

### Retrieval and preprocessing of mNGS data

mNGS data from fecal samples of 123 liver cirrhosis patient samples and 114 healthy control samples were derived from [14]. Across all samples, sequencing reads were examined as single-end. For samples with multiple sequencing runs, the sequencing reads across the runs were merged.

### Train-test split

In order to properly validate our results given our limited number of samples we performed ten-fold cross-validation. To that effect, we created ten different folds assigning 90% of the samples to the training and 10% in the test sets. Each fold consisted of liver cirrhosis patients and healthy control samples.

### Identification of kmers in each sample

For each sample, kmers of sixteen bp length were extracted using the Jellyfish package [6]. If a kmer appeared only once in a sample it was discarded from downstream sequencing analysis as a potential sequencing error.

### Derivation of frequentmers

We defined two groups, the first consisted of only healthy control samples and the second consisted only of liver cirrhosis patient samples. Frequentmers of recurrency r were defined as kmers that appeared in a minimum of r samples of the same group and were absent from every sample of the other group. To avoid over-fitting, the extraction of frequentmers was performed for each fold separately.

Identification of HBV or high alcohol consumption associated frequentmers was performed by analyzing the frequentmers present in HBV-positive samples or samples of individuals with high alcohol consumption.

To measure if the frequentmer profile between patient samples that were HBV positive or had high alcoholic intake differed from other patient samples we estimated the jaccard index based on the number of shared frequentmers between patient samples and examined the jaccard index distribution across all pairs in the two groups using paired t-tests.

### Frequentmer analysis

Recurrency thresholds of zero, three, five, ten and fifteen samples were examined. Analyses were performed independently for each recurrency threshold. For each recurrency threshold, results were averaged across the ten folds.

Majority voting, if it was found in more healthy control samples or more liver cirrhosis samples in the test set, was used to classify frequentmers found in the test set and calculate Mann–Whitney U statistic.

### Principal component analysis

To examine if frequentmers can linearly separate the liver cirrhosis patient samples from the healthy control samples we implemented principal Component Analysis with 90 components. However, the majority of the variance is captured by the first 20 components. The first three principal components were used to visually inspect the separation of the patient and the control samples. Principal Component Analysis was performed with the scikit-learn package [39].

### Classification models

Logistic regression was performed using healthy control and liver cirrhosis frequentmers as features with the scikit-learn package, using the parameters: penalty: Ridge (L2), max_iter: 2000 and C (the inverse regularization strength): 0.01. For each frequentmer, the coefficient score was derived and distribution histograms were generated for healthy control and liver cirrhosis frequentmers separately. The XGB-boost classification model was generated using the package from https://github.com/dmlc/xgboost with the parameters: max_depth = 11, gamma = 0.3, eta = 0.2, alpha = 6 [23].

To examine how the number of frequentmers used to train the logistic regression model affected the performance of the model, we used the absolute value of the logistic regression coefficients in the training set to re-train a model with the same sample split into training and test sets. The number of features examined ranged between 25 and 1,000 and performance was measured with the AUC score of each model. This process was repeated separately for each fold from which we derived the mean AUC score and confidence intervals across the ten folds.

### Frequentmer identification in microorganisms

Kraken2 taxonomic classification [40] using the standard reference database was performed for the reads containing the frequentmers with the highest and lowest coefficients from the logistic regression model. The standard reference database was built using adjusted parameters –kmer-len 16 –minimizer-len 15 –minimizer-spaces 3. An alluvial plot/sankey diagram was generated using Pavian [41]. Using the taxonomy labels generated from Kraken2, Bracken was performed to produce estimates of species- and genus- level abundance of each species [42]. The KrakenTools suite was used to calculate statistics and format the output from Bracken for visualization with Krona [43]. Krona was used to generate an RSF display that visualizes the Bracken output of the species- and genus- level relative abundance.

### Supplementary Information

**Additional file 1:** **Supplementary Figure 1.** Number of frequentmers detected as a function of the recurrency threshold. Recurrency thresholds of five to twenty samples were examined. Results shown for: A. healthy control and liver cirrhosis frequentmers, B. healthy control frequentmers, C. liver cirrhosis frequentmers. **Supplementary Figure 2.** As the recurrency threshold increases a larger proportion of frequentmers are patient frequentmers. Frequentmer ratio was defined as the ratio of patient frequentmers over healthy control and patient frequentmers. Values are averaged over ten folds. 99th percentile confidence intervals are shown. **Supplementary Figure 3.** Number of frequentmers observed in the test set. Sample recurrency of: A. 5, B. 10, C. 15, D. 20. Pink color represents healthy control frequentmers and purple represents liver cirrhosis frequentmers. All comparisons were statistically significant (Mann-Whitney U tests, *p*-value<0.0001). **Supplementary Figure 4.** The subset of frequentmers that are only found in HBV-positive patients. Recurrency threshold of: A: 5, B. 10, C. 15, D. 20 samples. **Supplementary Figure 5.** The subset of frequentmers that are only found in patients that had high alcohol intake. Recurrency threshold of: A: 5, B. 10, C. 15, D. 20 samples. **Supplementary Figure 6.** Logistic regression classification model ROC curve of liver cirrhosis and healthy control samples. Sample recurrency of A: 5, B. 10, C. 15, D. 20. Blue line represents the mean score, green lines represent the different folds and the gray area represents confidence intervals. **Supplementary Figure 7.** Histogram displaying the logistic regression coefficients. Sample recurrency of: A: 5, B. 10, C. 15, D. 20. **Supplementary Figure 8.** Ranked most important features by absolute coefficient score. A. Number of most important healthy control and patient frequentmers. B. Frequentmer ratio for most important healthy control and patient frequentmers. Frequentmer ratio is defined as the number of patient frequentmers over total frequentmers detected. Results shown for recurrency of fifteen. **Supplementary Figure 9.** XGBoost classification model ROC curve of liver cirrhosis and healthy control samples. Sample recurrency of A: 5bp, B. 10, C. 15, D. 20. Blue line represents the mean score, green lines represent the different folds and the gray area represents confidence intervals.

## Data Availability

The datasets analyzed during the current study are publicly available at the ENA Browser from ERP005860, [https://www.ebi.ac.uk/ena/browser/view/ERP005860]. All the associated code used for the generation of figures and presentation of data throughout the manuscript is deposited on GitHub at the following link: https://github.com/Georgakopoulos-Soares-lab/frequentmer_analysis

## References

[1] Lee S, Huang H, Zelen M (2004). Early detection of disease and scheduling of screening examinations. Stat Methods Med Res.

[2] Rahman A, Hallgrímsdóttir I, Eisen M, Pachter L. Association mapping from sequencing reads using -mers. Elife. 2018;7. 10.7554/eLife.32920.001. 10.7554/eLife.32920 PMC6044908 29897334

[3] Rhie A, Walenz BP, Koren S, Phillippy AM (2020). Merqury: reference-free quality, completeness, and phasing assessment for genome assemblies. Genome Biol.

[4] Yang Z, Li H, Jia Y, Zheng Y, Meng H, Bao T (2020). Intrinsic laws of k-mer spectra of genome sequences and evolution mechanism of genomes. BMC Evol Biol.

[5] Bussi Y, Kapon R, Reich Z (2021). Large-scale k-mer-based analysis of the informational properties of genomes, comparative genomics and taxonomy. PLoS One.

[6] Marçais G, Kingsford C (2011). A fast, lock-free approach for efficient parallel counting of occurrences of k-mers. Bioinformatics.

[7] Bushnell B, Rood J, Singer E (2017). BBMerge - accurate paired shotgun read merging via overlap. PLoS One.

[8] Acquisti C, Poste G, Curtiss D, Kumar S (2007). Nullomers: really a matter of natural selection?. PLoS One.

[9] Georgakopoulos-Soares I, Yizhar-Barnea O, Mouratidis I, Hemberg M, Ahituv N (2021). Absent from DNA and protein: genomic characterization of nullomers and nullpeptides across functional categories and evolution. Genome Biol.

[10] Koulouras G, Frith MC (2021). Significant non-existence of sequences in genomes and proteomes. Nucleic Acids Res.

[11] Mouratidis I, Chan CSY, Chantzi N, Tsiatsianis GC, Hemberg M, Ahituv N (2023). Quasi-prime peptides: identification of the shortest peptide sequences unique to a species. NAR Genom Bioinform.

[12] Liu YB, Chen MK (2022). Epidemiology of liver cirrhosis and associated complications: current knowledge and future directions. World J Gastroenterol.

[13] GBD 2017 Cirrhosis Collaborators (2020). The global, regional, and national burden of cirrhosis by cause in 195 countries and territories, 1990–2017: a systematic analysis for the Global Burden of Disease Study 2017. Lancet Gastroenterol Hepatol.

[14] Qin N, Yang F, Li A, Prifti E, Chen Y, Shao L (2014). Alterations of the human gut microbiome in liver cirrhosis. Nature.

[15] Marcos-Zambrano LJ, Karaduzovic-Hadziabdic K, Loncar Turukalo T, Przymus P, Trajkovik V, Aasmets O (2021). Applications of machine learning in human microbiome studies: a review on feature selection, biomarker identification, disease prediction and treatment. Front Microbiol.

[16] Irber L, Brooks PT, Reiter T, Tessa Pierce-Ward N, Hera MR, Koslicki D, et al. Lightweight compositional analysis of metagenomes with FracMinHash and minimum metagenome covers. bioRxiv. 2022 . p. 2022.01.11.475838. Available from: https://www.biorxiv.org/content/10.1101/2022.01.11.475838v2.abstract. Cited 2023 Oct 27.

[17] Reiter TE, Irber L, Gingrich AA, Haynes D, Tessa Pierce-Ward N, Brooks PT, et al. Meta-analysis of metagenomes via machine learning and assembly graphs reveals strain switches in Crohn’s disease. bioRxiv. 2022. p. 2022.06.30.498290. Available from: https://www.biorxiv.org/content/10.1101/2022.06.30.498290v1.abstract. Cited 2023 Oct 27.

[18] Koohi-Moghadam M, Borad MJ, Tran NL, Swanson KR, Boardman LA, Sun H (2019). MetaMarker: a pipeline for de novo discovery of novel metagenomic biomarkers. Bioinformatics.

[19] Wang Y, Fu L, Ren J, Yu Z, Chen T, Sun F. Identifying group-specific sequences for microbial communities using long k-mer sequence signatures. Front Microbiol. 2018;9. Available from: https://www.ncbi.nlm.nih.gov/pmc/articles/PMC5943621/. Cited 2023 Oct 27. 10.3389/fmicb.2018.00872 PMC5943621 29774017

[20] Georgakopoulos-Soares I, Barnea OY, Mouratidis I, Bradley R, Easterlin R, Chan C, et al. Leveraging sequences missing from the human genome to diagnose cancer. medRxiv. 2021. 10.1038/s43856-025-01067-3 PMC12371106 40841759

[21] Scaglione S, Kliethermes S, Cao G, Shoham D, Durazo R, Luke A (2015). The epidemiology of cirrhosis in the United States: a population-based study. J Clin Gastroenterol.

[22] Trivedi HD, Tapper EB (2018). Interventions to improve physical function and prevent adverse events in cirrhosis. Gastroenterol Rep.

[23] Chen T, Guestrin C. XGBoost: a scalable tree boosting system. In: Proceedings of the 22nd ACM SIGKDD International Conference on Knowledge Discovery and Data Mining. New York: Association for Computing Machinery; 2016. p. 785–94. (KDD ’16).

[24] Dong TS, Katzka W, Lagishetty V, Luu K, Hauer M, Pisegna J (2020). A microbial signature identifies advanced fibrosis in patients with chronic liver disease mainly due to NAFLD. Sci Rep.

[25] Liu Y, Wang JY, Jiang W (2013). An increasing prominent disease of Klebsiella pneumoniae liver abscess: etiology, diagnosis, and treatment. Gastroenterol Res Pract.

[26] Hu W, Gao W, Liu Z, Fang Z, Wang H, Zhao J (2022). Specific strains of ameliorate nonalcoholic fatty liver disease in mice in association with gut microbiota regulation. Nutrients.

[27] Chen Y, Liu P, Liu R, Hu S, He Z, Dong G, et al. Comprehensive strain-level analysis of the gut microbe faecalibacterium prausnitzii in patients with liver cirrhosis. mSystems. 2021;6(4):e0077521. 10.1128/mSystems.00775-21 PMC8407477 34342541

[28] Yuan J, Chen C, Cui J, Lu J, Yan C, Wei X (2019). Fatty liver disease caused by high-alcohol-producing Klebsiella pneumoniae. Cell Metab.

[29] Kellner MJ, Koob JG, Gootenberg JS, Abudayyeh OO, Zhang F (2019). SHERLOCK: nucleic acid detection with CRISPR nucleases. Nat Protoc.

[30] Loose M, Malla S, Stout M (2016). Real-time selective sequencing using nanopore technology. Nat Methods.

[31] Helmink BA, Khan MAW, Hermann A, Gopalakrishnan V, Wargo JA (2019). The microbiome, cancer, and cancer therapy. Nat Med.

[32] Romano S, Savva GM, Bedarf JR, Charles IG, Hildebrand F, Narbad A (2021). Meta-analysis of the Parkinson’s disease gut microbiome suggests alterations linked to intestinal inflammation. NPJ Parkinsons Dis.

[33] Fan Y, Pedersen O (2021). Gut microbiota in human metabolic health and disease. Nat Rev Microbiol.

[34] De Luca F, Shoenfeld Y (2019). The microbiome in autoimmune diseases. Clin Exp Immunol.

[35] Franzosa EA, Sirota-Madi A, Avila-Pacheco J, Fornelos N, Haiser HJ, Reinker S (2019). Gut microbiome structure and metabolic activity in inflammatory bowel disease. Nat Microbiol.

[36] Whiteside SA, Razvi H, Dave S, Reid G, Burton JP (2015). The microbiome of the urinary tract–a role beyond infection. Nat Rev Urol.

[37] Natalini JG, Singh S, Segal LN (2023). The dynamic lung microbiome in health and disease. Nat Rev Microbiol.

[38] Honda K, Littman DR (2012). The microbiome in infectious disease and inflammation. Annu Rev Immunol.

[39] Pedregosa F, Varoquaux G, Gramfort A, Michel V, Thirion B, Grisel O, et al. Scikit-learn: machine learning in Python. J Mach Learn Res.

[40] Wood DE, Lu J, Langmead B (2019). Improved metagenomic analysis with Kraken 2. Genome Biol.

[41] Breitwieser FP, Salzberg SL (2020). Pavian: interactive analysis of metagenomics data for microbiome studies and pathogen identification. Bioinformatics.

[42] Lu J, Rincon N, Wood DE, Breitwieser FP, Pockrandt C, Langmead B (2022). Metagenome analysis using the Kraken software suite. Nat Protoc.

[43] Ondov BD, Bergman NH, Phillippy AM (2011). Interactive metagenomic visualization in a web browser. BMC Bioinformatics.

