# Supplementary material for: Frequentmers - a novel way to look at metagenomic next generation sequencing data and an application in detecting liver cirrhosis
Source: BMC Genomics. 2023 Dec 12;24:768. doi: 10.1186/s12864-023-09861-w (PMC10714505; doi:10.1186/s12864-023-09861-w)
Supplement: Supplementary file 1 — Additional file 1: Supplementary Figure 1. Number of frequentmers detected as a function of the recurrency threshold. Recurrency thresholds of five to twenty samples were examined. Results shown for: A. healthy control and liver cirrhosis frequentmers, B. healthy control frequentmers, C. liver cirrhosis frequentmers. Supplementary Figure 2. As the recurrency threshold increases a larger proportion of frequentmers are patient frequentmers. Frequentmer ratio was defined as the ratio of patient frequentmers over healthy control and patient frequentmers. Values are averaged over ten folds. 99th percentile confidence intervals are shown. Supplementary Figure 3. Number of frequentmers observed in the test set. Sample recurrency of: A. 5, B. 10, C. 15, D. 20. Pink color represents healthy control frequentmers and purple represents liver cirrhosis frequentmers. All comparisons were statistically significant (Mann-Whitney U tests, p-value<0.0001). Supplementary Figure 4. The subset of frequentmers that are only found in HBV-positive patients. Recurrency threshold of: A: 5, B. 10, C. 15, D. 20 samples. Supplementary Figure 5. The subset of frequentmers that are only found in patients that had high alcohol intake. Recurrency threshold of: A: 5, B. 10, C. 15, D. 20 samples. Supplementary Figure 6. Logistic regression classification model ROC curve of liver cirrhosis and healthy control samples. Sample recurrency of A: 5, B. 10, C. 15, D. 20. Blue line represents the mean score, green lines represent the different folds and the gray area represents confidence intervals. Supplementary Figure 7. Histogram displaying the logistic regression coefficients. Sample recurrency of: A: 5, B. 10, C. 15, D. 20. Supplementary Figure 8. Ranked most important features by absolute coefficient score. A. Number of most important healthy control and patient frequentmers. B. Frequentmer ratio for most important healthy control and patient frequentmers. Frequentmer ratio is defined as the number of [file 12864_2023_9861_MOESM1_ESM.docx]

**Supplementary Figures**

A


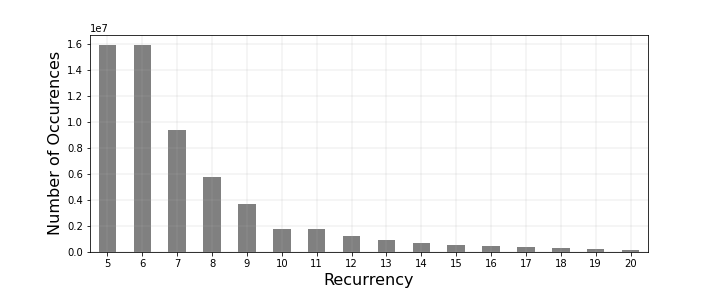


B


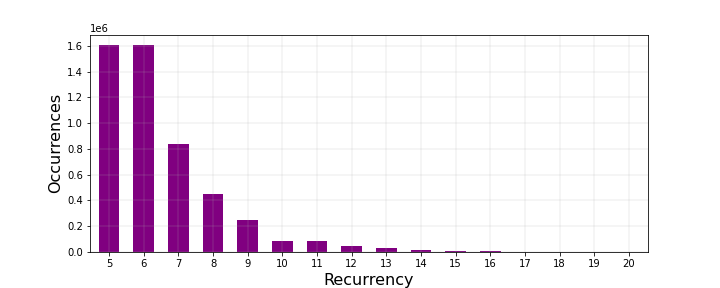


C


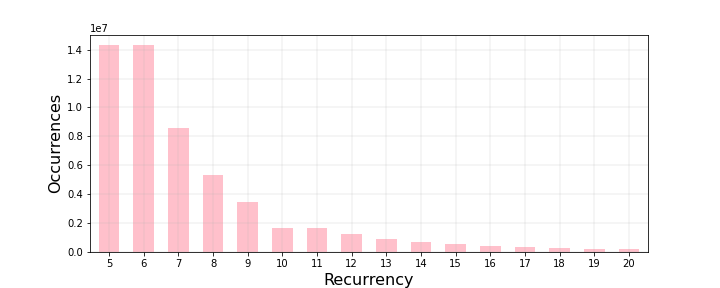


**Supplementary Figure 1: Number of frequentmers detected as a function of the recurrency threshold.** Recurrency thresholds of five to twenty samples were examined. Results shown for: **A.** healthy control and liver cirrhosis frequentmers, **B.** healthy control frequentmers, **C.** liver cirrhosis frequentmers.


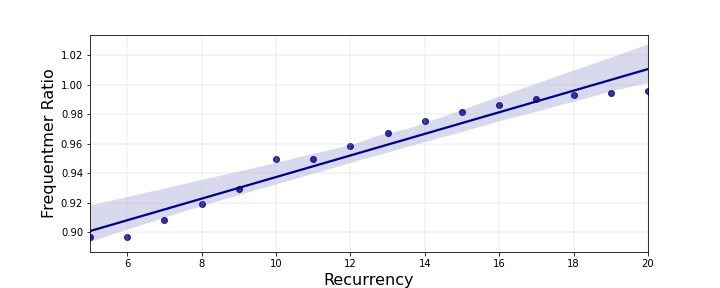


**Supplementary Figure 2: As the recurrency threshold increases a larger proportion of frequentmers are patient frequentmers.** Frequentmer ratio was defined as the ratio of patient frequentmers over healthy control and patient frequentmers. Values are averaged over ten folds. 99th percentile confidence intervals are shown.

A B


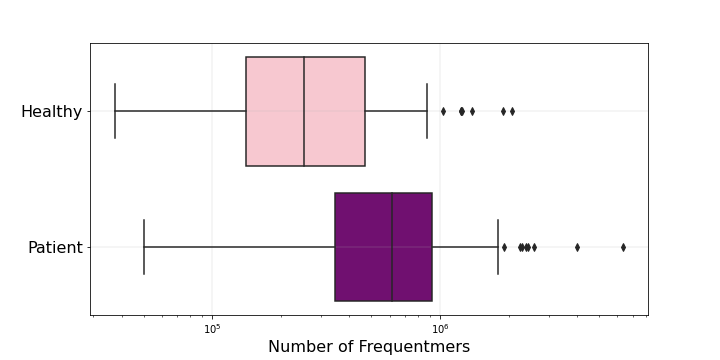

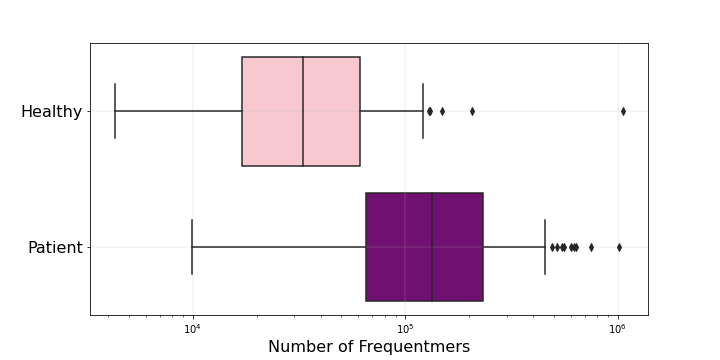


C D


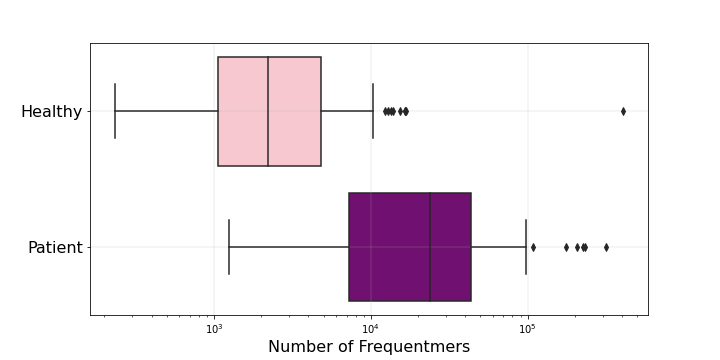

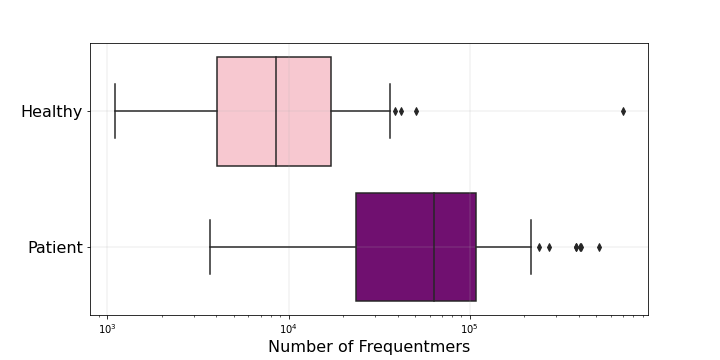


**Supplementary Figure 3: Number of frequentmers observed in the test set.** Sample recurrency of: **A.** 5, **B.** 10, **C.** 15, **D.** 20. Pink color represents healthy control frequentmers and purple represents liver cirrhosis frequentmers. All comparisons were statistically significant (Mann-Whitney U tests, p-value<0.0001).

A B


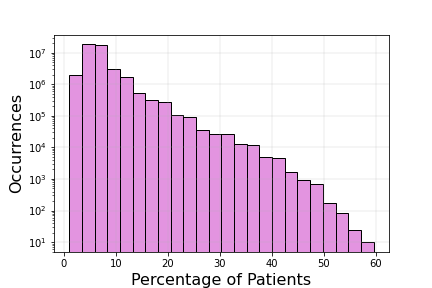

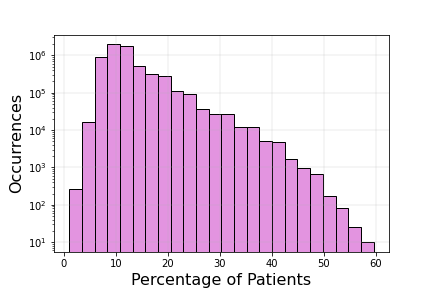


C D


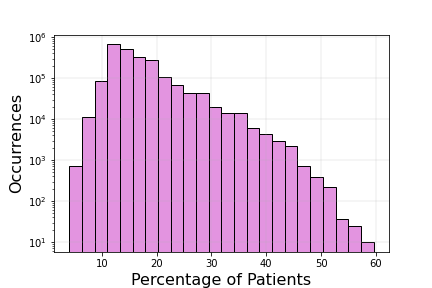

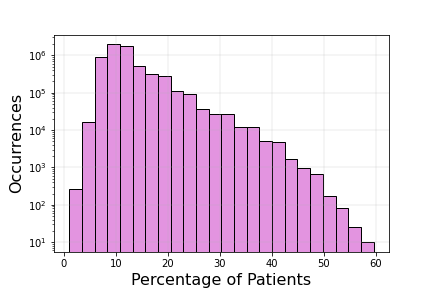


**Supplementary Figure 4: The subset of frequentmers that are only found in HBV-positive patients.** Recurrency threshold of: **A:** 5, **B.** 10, **C.** 15, **D.** 20 samples.

A B


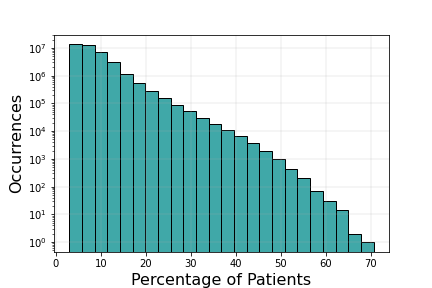

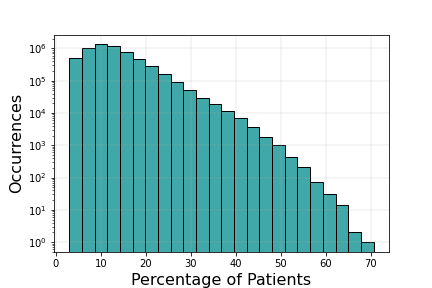


C D


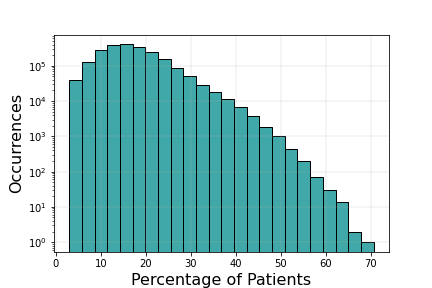

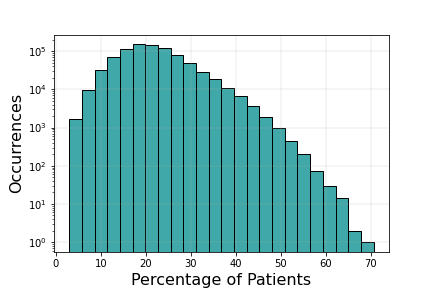


**Supplementary Figure 5: The subset of frequentmers that are only found in patients that had high alcohol intake.** Recurrency threshold of: **A:** 5, **B.** 10, **C.** 15, **D.** 20 samples.

A B


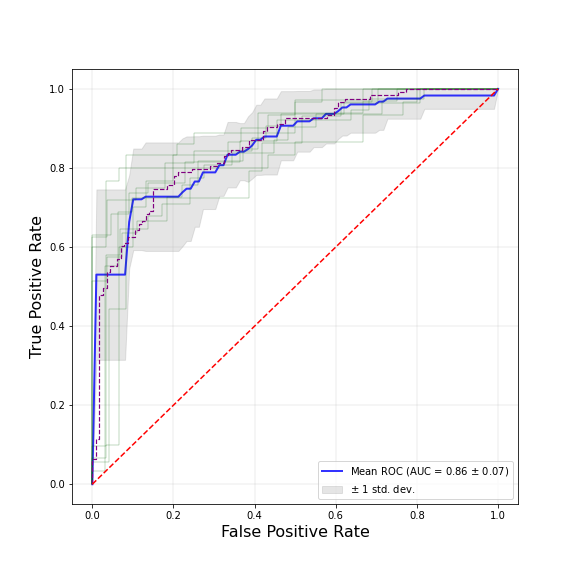

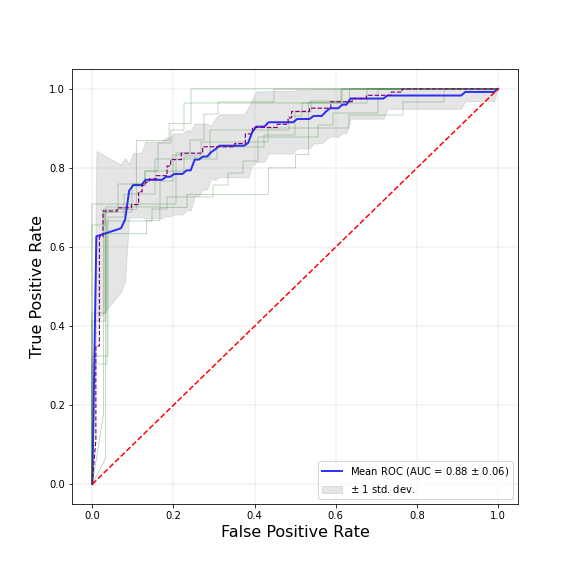


C D


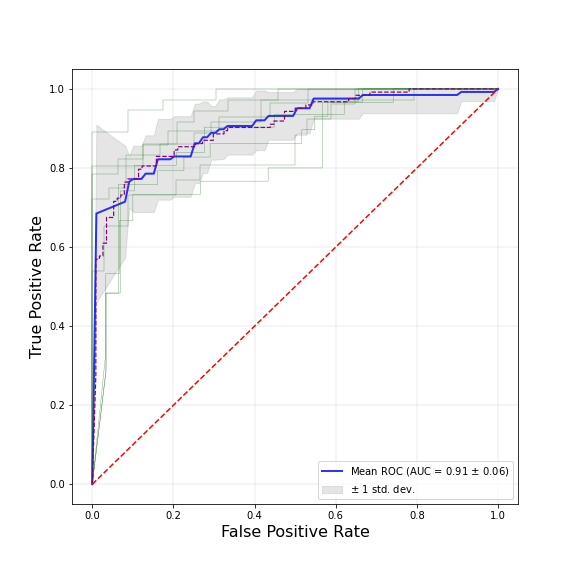

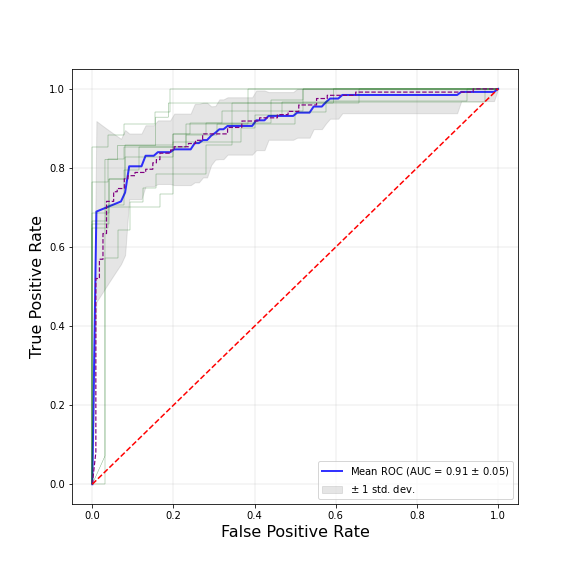


**Supplementary Figure 6: Logistic regression classification model ROC curve of liver cirrhosis and healthy control samples.** Sample recurrency of **A:** 5, **B.** 10, **C.** 15, **D.** 20. Blue line represents the mean score, green lines represent the different folds and the gray area represents confidence intervals.

A B


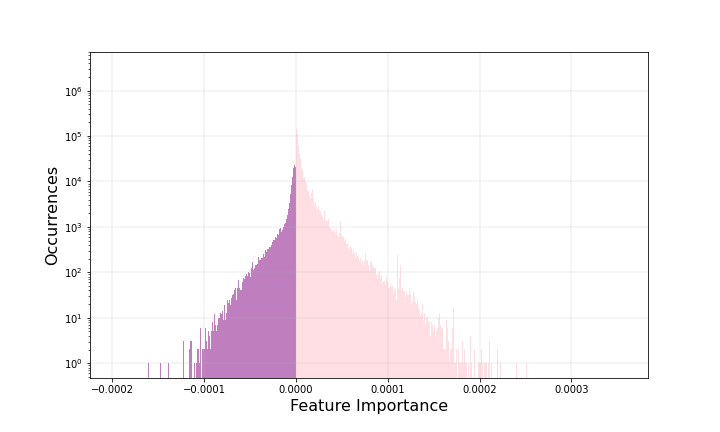

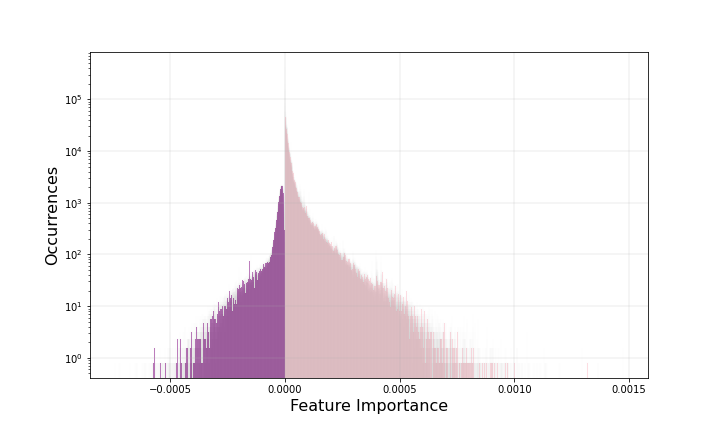


C D


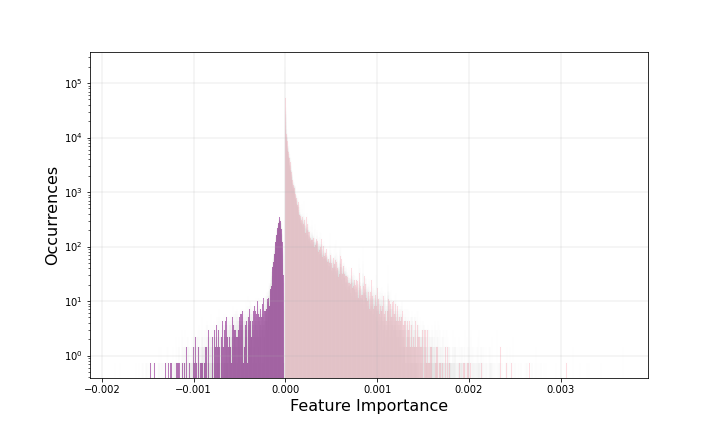

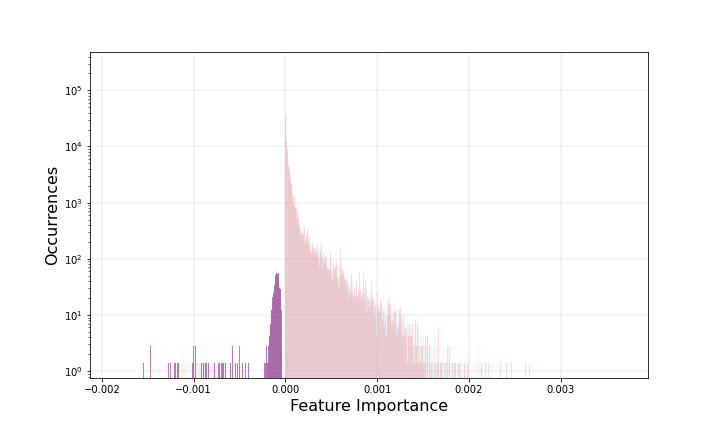

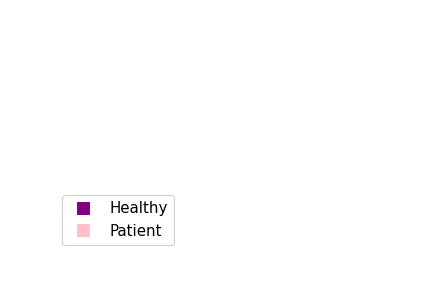


**Supplementary Figure 7: Histogram displaying the logistic regression coefficients.** Sample recurrency of: **A:** 5, **B.** 10, **C.** 15, **D.** 20.

**
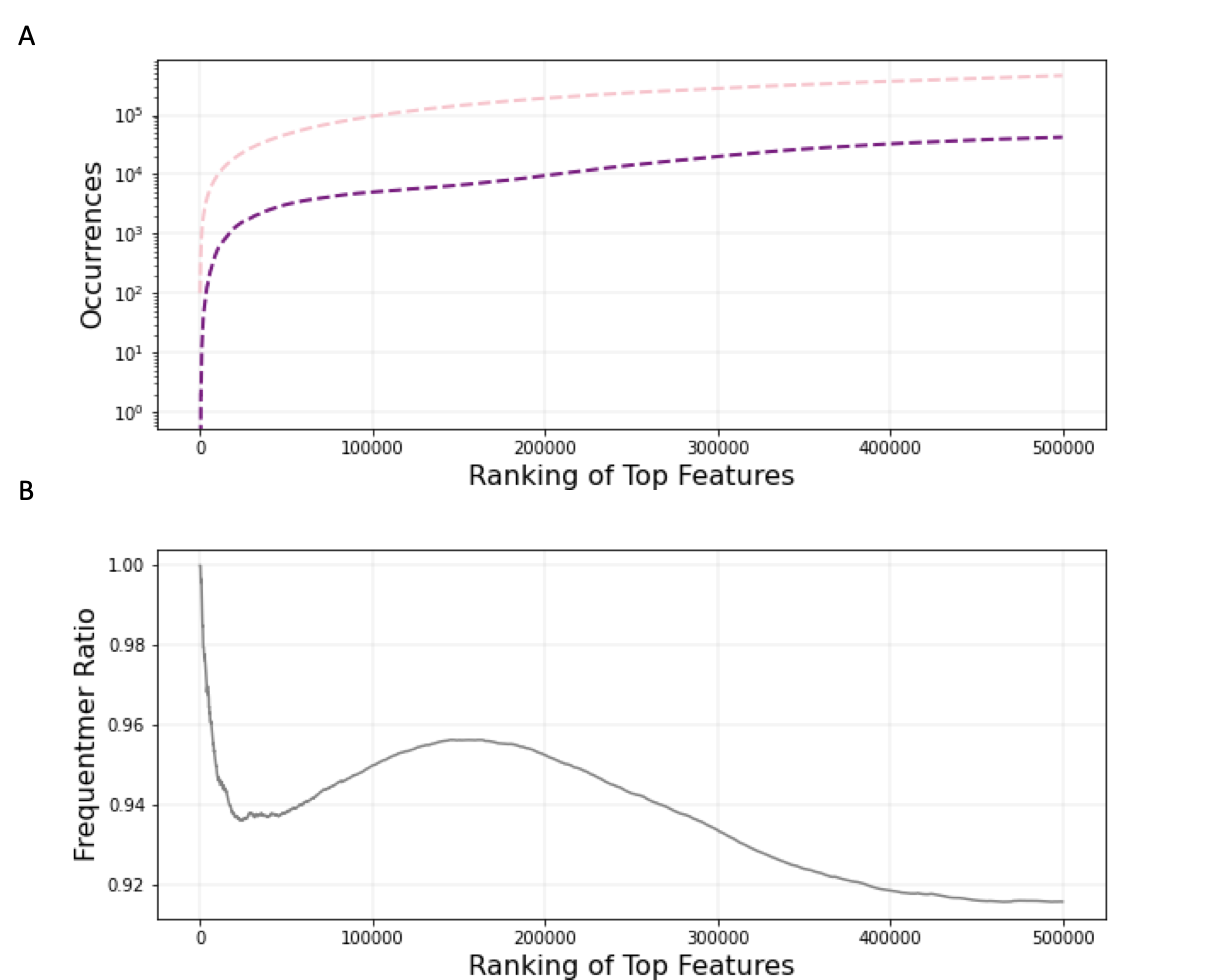
**


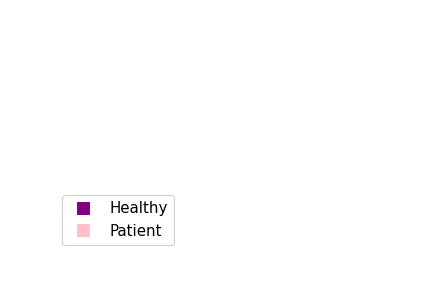


**Supplementary Figure 8: Ranked most important features by absolute coefficient score. A.** Number of most important healthy control and patient frequentmers. **B.** Frequentmer ratio for most important healthy control and patient frequentmers. Frequentmer ratio is defined as the number of patient frequentmers over total frequentmers detected. Results shown for recurrency of fifteen.

A B

**
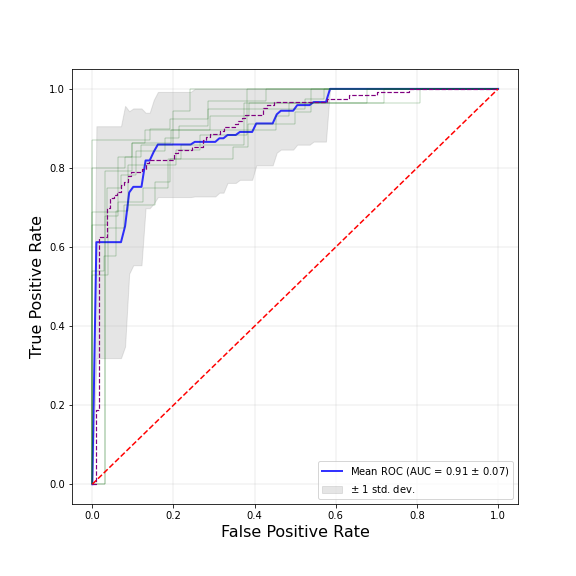

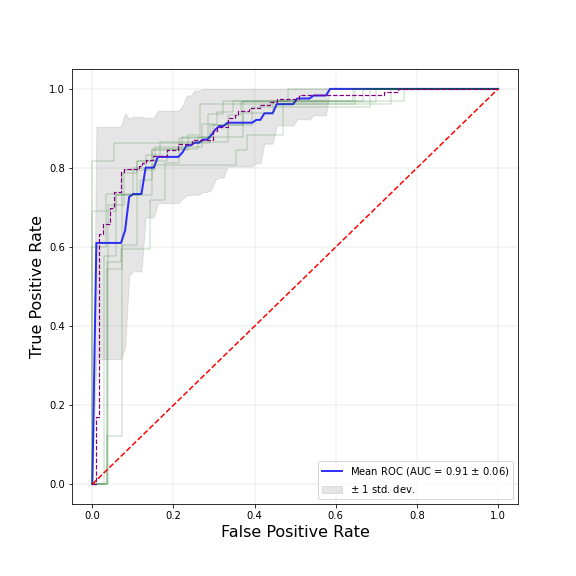
**

C D

**
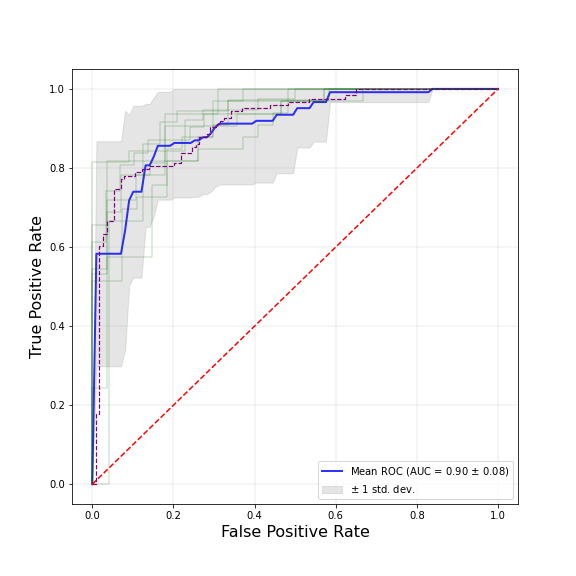

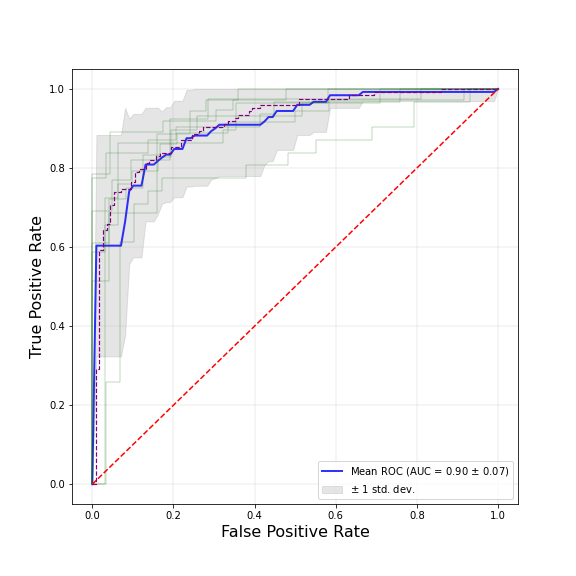
**

**Supplementary Figure 9: XGBoost classification model ROC curve of liver cirrhosis and healthy control samples.** Sample recurrency of **A:** 5bp, **B.** 10, **C.** 15, **D.** 20. Blue line represents the mean score, green lines represent the different folds and the gray area represents confidence intervals.
